# Flexible Piezoelectric Sensor-Based Gait Recognition

**DOI:** 10.3390/s18020468

**Published:** 2018-02-05

**Authors:** Youngsu Cha, Hojoon Kim, Doik Kim

**Affiliations:** 1 Center for Robotics Research, Korea Institute of Science and Technology, Seoul 02792, Korea; hojun5266@korea.ac.kr (H.K.); doikkim@kist.re.kr (D.K.); 2 School of Electrical Engineering, Korea University, Seoul 02841, Korea

**Keywords:** flexible sensor, gait recognition, piezoelectric material, wearable

## Abstract

Most motion recognition research has required tight-fitting suits for precise sensing. However, tight-suit systems have difficulty adapting to real applications, because people normally wear loose clothes. In this paper, we propose a gait recognition system with flexible piezoelectric sensors in loose clothing. The gait recognition system does not directly sense lower-body angles. It does, however, detect the transition between standing and walking. Specifically, we use the signals from the flexible sensors attached to the knee and hip parts on loose pants. We detect the periodic motion component using the discrete time Fourier series from the signal during walking. We adapt the gait detection method to a real-time patient motion and posture monitoring system. In the monitoring system, the gait recognition operates well. Finally, we test the gait recognition system with 10 subjects, for which the proposed system successfully detects walking with a success rate over 93%.

## 1. Introduction

Recent advancements in wearable sensors have promoted major scientific and technological developments in the field of human activity recognition [1,2]. Wearable sensors, for this purpose, have significant benefits over external sensors in terms of privacy, pervasiveness, and complexity [1]. Most people would be averse to being continuously monitored by external cameras [1,3]. It is also difficult to obtain full body imagery all the time. Moreover, image processing to recognize human activities is computationally complex.

Gait recognition and analysis are one of the most important research areas, because walking is a common human physical activity [2,4]. The gait recognition system can be utilized for therapeutic and diagnostic applications [5,6]. In addition, the sensing part can be combined to gait assistant system [7,8]. Various wearable sensors, such as inertial measurement units (IMU), pressure sensors, force sensitive resistors, accelerometers, and gyroscopes, are utilized for gait recognition and analysis [2,4,9]. These sensors are positioned on hips, thighs, knees, shanks, and feet in the lower body; they measure angles and contacts during walking [4]. For example, a piezoelectric gyroscope on the thigh of one leg has been used for detecting stride length and walking velocity [10]. Piezoresistive accelerometers have been studied in ambulatory monitoring [11]. A real-time gait phase detection system with a gyroscope and force sensitive resistors installed inside a shoe insole has also been presented [12]. Bending type piezoelectric accelerometers have been utilized for footstep detection [13]. A method of measuring joint angle for gait using a combination of accelerometers and gyroscopes has been presented [14]. A step counting method, using tri-axial accelerometers on the ankles, thigh, and waist has been proposed [15]. In most research, wearable sensors have been tightly attached to the body for accurate sensing. However, tight attachment can cause inconvenience for users.

In this paper, we introduce a flexible piezoelectric sensor-based gait recognition system available in loose clothing. The flexible sensor is made of a piezoelectric material: polyvinylidene fluoride (PVDF) [16,17,18]. PVDF is one of the most flexible piezoelectric materials, outputting voltage signals when it experiences strain [19,20,21]. Four flexible piezoelectric sensors are attached to the knee and hip parts of the loose clothing. We measure the sensor outputs during human walking by analyzing the raw signals, signal processed values, and fast Fourier transform (FFT) magnitudes. Furthermore, we demonstrate a gait detection system based on the outputs of the flexible piezoelectric sensors. Specifically, we add the gait detection method to the real-time patient motion and posture monitoring system reported in [22]. The integration of the gait detection to the monitoring system presents that the proposed method can be combined as other posture detections or system. In addition, the monitoring system can have an important update about gait detection of patients through the integration. The monitoring system recognizes five postures including walking and eight transitions between postures. We show the operation of the monitoring system in a demo scenario. Additionally, we conduct user tests of gait detection in the monitoring system.

This paper is organized as follows. In Section 2, we introduce the experimental setup of the flexible piezoelectric sensor, the module for collecting the sensor’s signal, and walking test on a treadmill. In Section 3, we present an investigation of the sensor outputs during the walking motion. The decision method for gait recognition using the sensor outputs during walking is described in Section 4. In Section 5, we present the demonstration of gait recognition with a real-time patient motion and posture monitoring system. The conclusions are summarized in the final section.

## 2. Experimental Setup

**Figure 1:**
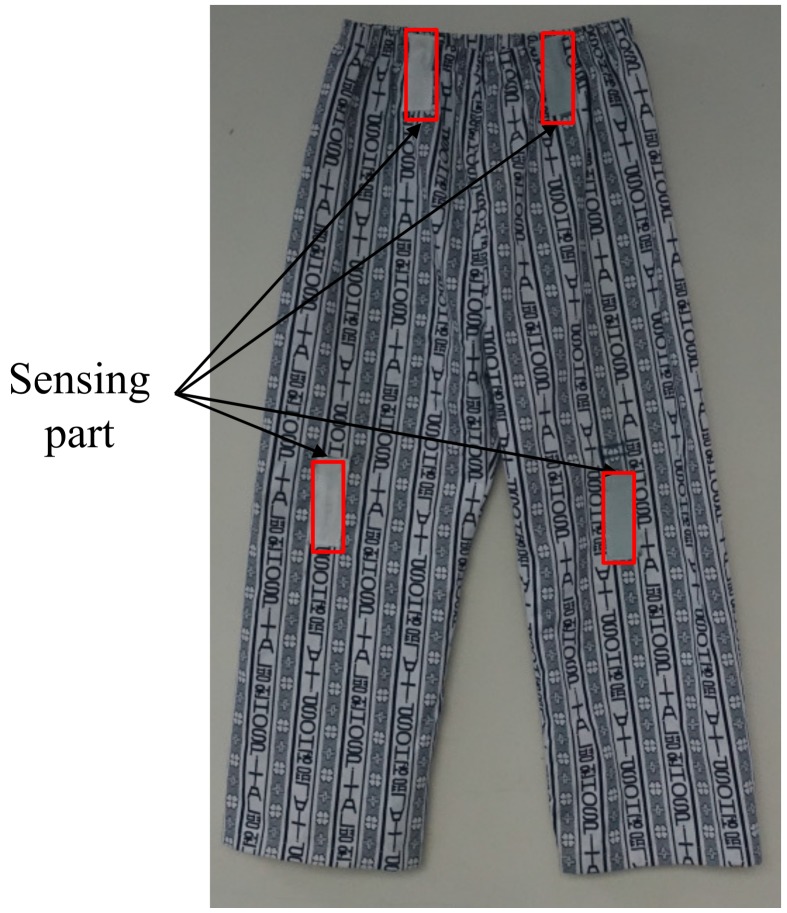
Patient clothing for four flexible sensors.

We use similar patient cloth with flexible sensors as [22] for our experiments. In this work, the four sensors are embedded in both knee and hip parts on the patient’s pants (see Figure 1). The sensors are composed of PVDF, produced by Measurement Specialties, and Mylar [17,22]. A PVDF sheet is glued on a Mylar sheet with 3M DP460 epoxy. The sensor size is $\left(L\right)75\times \left(W\right)25\times \left(T\right)0.3\;{\mathrm{mm}}^{3}$. We sense electrical signals from the flexible sensors using conductive adhesive copper tape electrodes, produced by 3M, attached on both surface of PVDF. The copper electrodes are connected to a sensing module through wires. The voltages from the sensors, carried by the wires, are digitized using the analog-digital converters in the sensing module. The signals are then transmitted by Bluetooth wireless communication. The sensing frequency is approximately 100Hz.

A KTS 7500TS treadmill is utilized to test walking at standard speed. In the tests, a male student (age: 25 years, height: 170 cm, weight: 70 kg) wears the patient clothes with flexible sensors and walks on the treadmill. The test walking speed varies from 0.5 to 6km/h, which, if exceeded, requires running. When the student walks on the treadmill, we record the sensor outputs via the sensing system.

## 3. Sensor Output for Walking

**Figure 2:**
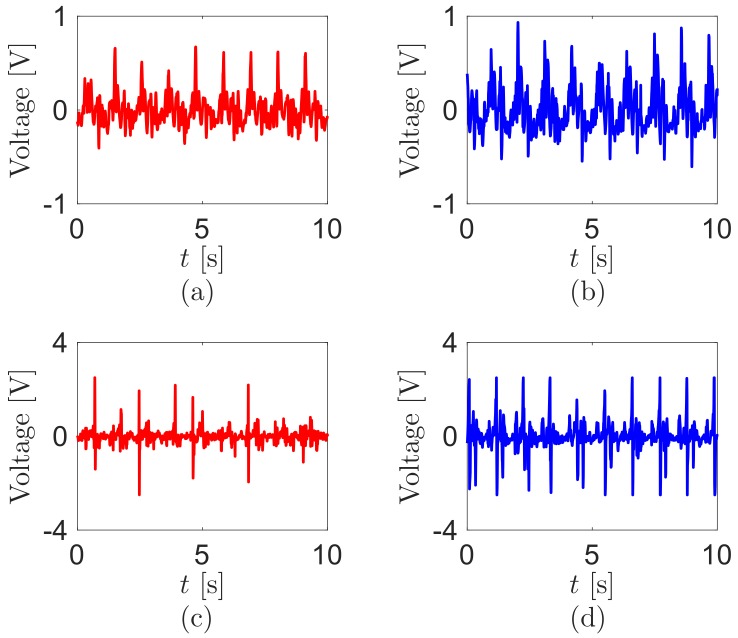
Sensor voltage outputs during walking at the (**a**) left hip; (**b**) right hip; (**c**) left knee; and (**d**) right knee. The test walking velocity is 4km/h.

We analyze the sensor outputs during walking. Figure 2 shows the voltage outputs from the sensors during walking at 4km/h on the treadmill. The signal amplitudes at both knee sensors are bigger than those from the hip sensors because of several peaks. However, all signal wave forms of the hip sensors are more periodic than the knee sensors. The differences can be attributed to the loose patient cloth. During walking, the knee has a larger bending angle motion than the hip [23]. With tight clothes, the electric signal from the piezoelectric sensor attached to the knee will be larger than the signal at the hip [24]. With the loose clothing of this test, the knee sensors do not perfectly capture the periodic motions of the knees during walking. Conversely, the hip signals are sensed well during walking, because the waist band of the pants fits more tightly. Thus, the hip sensors are attached more closely than the knee sensors.

**Figure 3:**
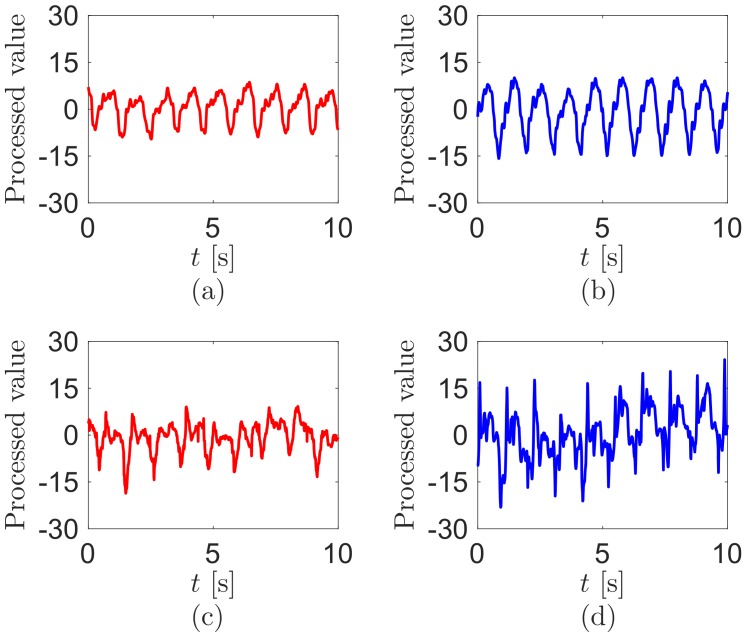
Signal processed values from the sensor outputs during walking at the (**a**) left hip; (**b**) right hip; (**c**) left knee; and (**d**) right knee. The test walking velocity is 4km/h.

**Figure 4:**
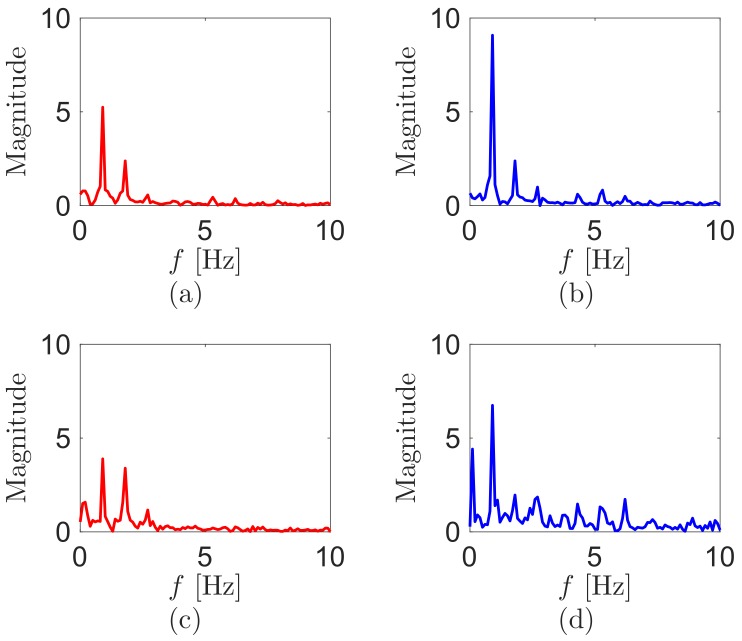
FFT magnitude spectra of the signal processed values from the sensor outputs during walking at the (**a**) left hip; (**b**) right hip; (**c**) left knee; and (**d**) right knee. The test walking velocity is 4km/h.

The sensor voltages correlate to the angular velocities of their bending [22]. We process data to acquire the bending motions from the sensor voltages, following the methods of [22]. Briefly, the sensor voltage outputs are processed through removing their voltage offsets and integrating as the time. We sense clearer periodic signals during walking (see Figure 3). Similarly, the processed values at the hip are more periodic than at the knee. To clarify the periodicity between the processed values at the hip and knee, we conduct FFT in MATLAB using the processed values. Figure 4 displays the FFT results from the signal processed values of Figure 3. The magnitudes of the main walking frequency in all FFT results are the largest. However, the results at the knee show that the higher harmonic frequencies still have big amplitudes.

## 4. Gait Recognition

**Figure 5:**
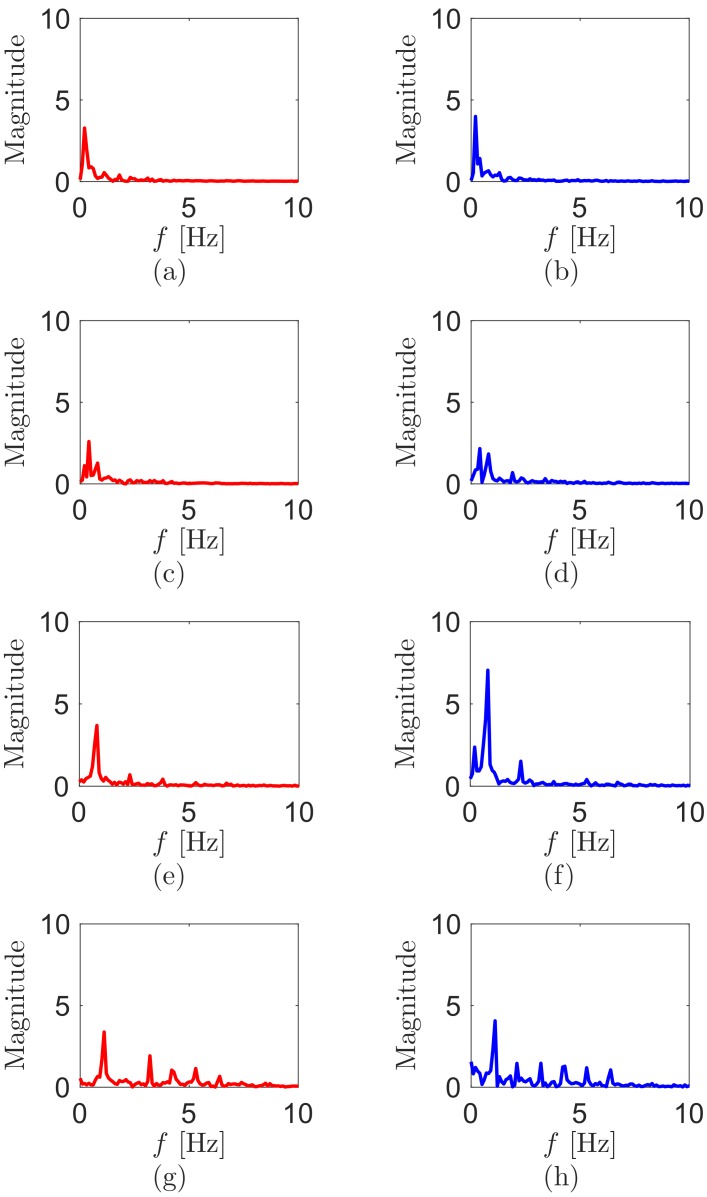
FFT magnitude spectra during walking of (**a**) left and (**b**) right hip at 0.5km/h, (**c**) left and (**d**) right hip at 1km/h, (**e**) left and (**f**) right hip at 3km/h, and (**g**) left and (**h**) right hip at 6km/h.

We decide to use only left and right hip sensors to recognize walking motion, based on the findings in Section 3. We perform additional tests at various walking speeds (0.5, 1, 2, 3, 4, 5 and 6km/h) on the treadmill. Figure 5 displays the FFT magnitude spectra at 0.5, 1, 3 and 6km/h. We can detect the main walking frequency by finding the biggest magnitude in each plot. We observe that the main walking frequencies increase with the walking speeds. The walking frequency as the speed of the treadmill is presented in Table 1. Herein, we focus on normal walking speed [25], which is approximately 1.1m/s (∼4km/h). At that walking speed, the main frequency is between 0.9 and 1Hz.

For detecting the walking frequency in real-time system, we use discrete time Fourier series instead of FFT to reduce computing time. In the same context, we obtain the series coefficients of the frequency, 1Hz, and the second harmonic. Specifically, the coefficients are obtained every half second. When the average of the two coefficients is over a decision value, we make a decision about the gait recognition. Herein, we select the decision value of 0.8 from preliminary tests. When we use the gait recognition method with the decision value, the detection rate is over 90% at 3–6km/h.

## 5. System Integration

**Figure 6:**
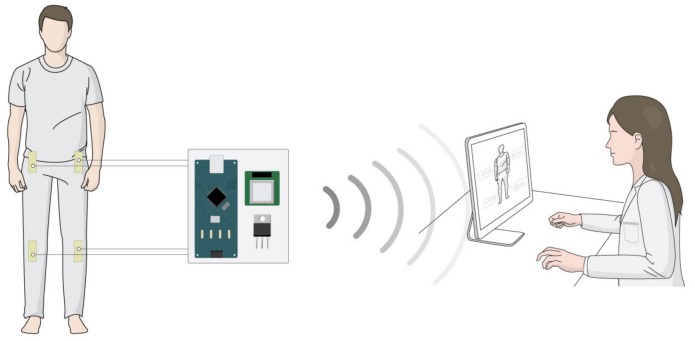
Experimental setup for testing the patient motion and posture monitoring system.

**Figure 7:**
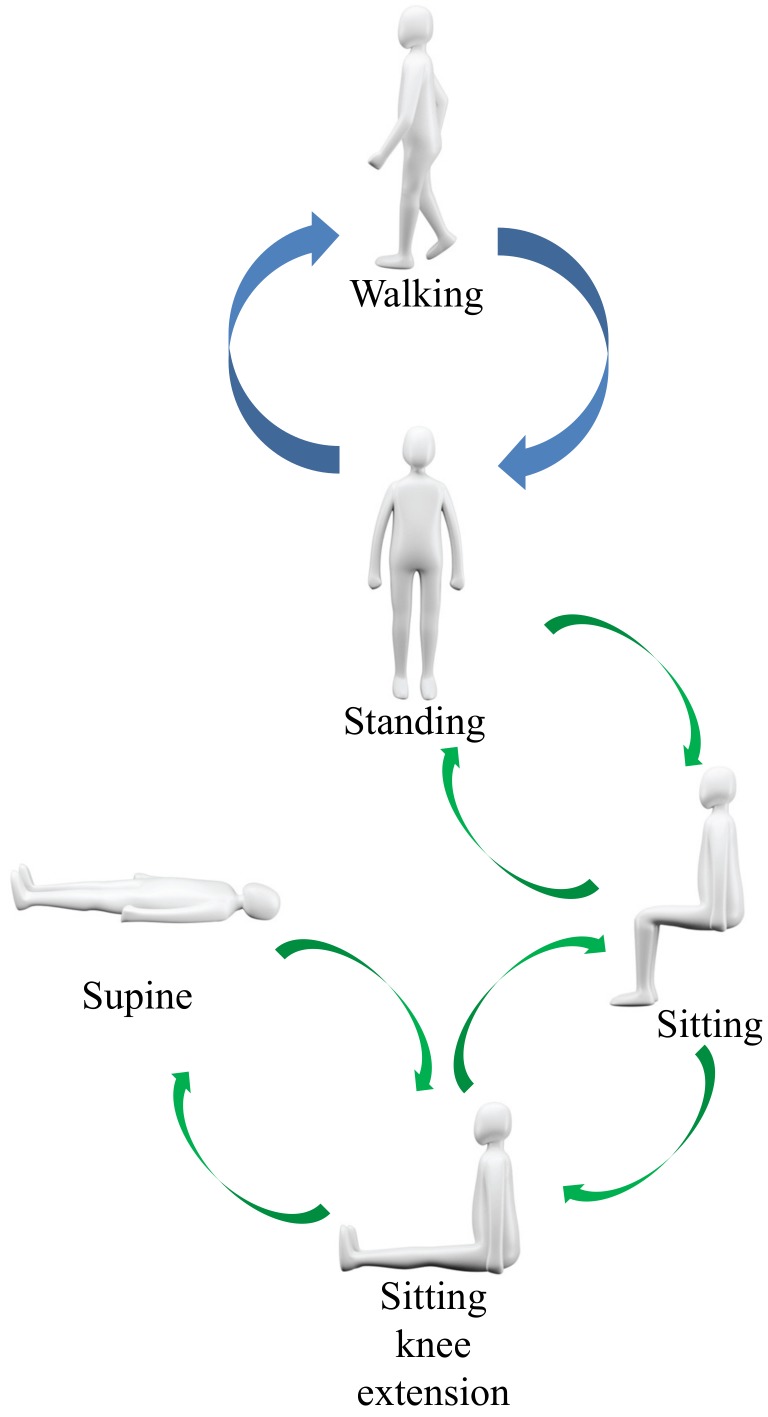
Flow chart for motion and posture decisions.

We adapt the gait detection to the patient monitoring system of [22]. The patient monitoring system was developed for motion and posture recognitions of patients; the gait detection method is also programmed for the system. Figure 6 displays the test condition for the patient motion and posture monitoring system. Specifically, the flexible sensor data is collected and processed in an external computer for monitoring; the monitoring program shows the decision about the motion and posture of the patient. In the monitoring program, we can detect a total of eight transitions and five postures. The five postures are (i) walking, (ii) standing, (iii) sitting, (iv) sitting knee extension, and (v) supine. We also predict the motions during the transitions between the postures (see Figure 7). To avoid incorrect operations, the walking is only transited from and to the standing pose. We note that the total four sensors’ data are monitored in the system, the two hip sensors’ data are utilized for the gait detection, and the two left sensors’ data are used for capturing the transitions between standing, sitting, sitting knee extension, and supine. We comment that the system follows [22] about the detailed procedures for the decision between the postures of standing, sitting, sitting knee extension, and supine.

**Figure 8:**
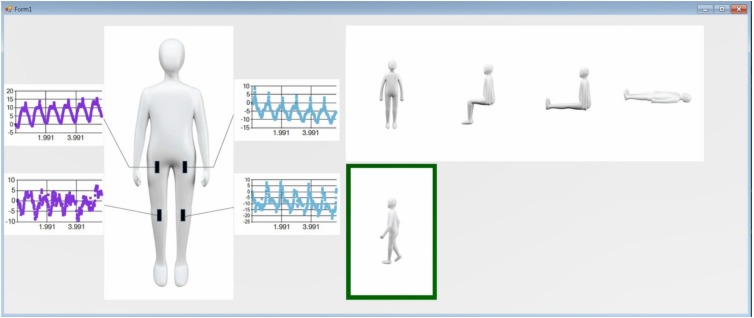
Captured computer screen showing patient walking detection and signal processed values from the four sensor outputs.

**Figure 9:**
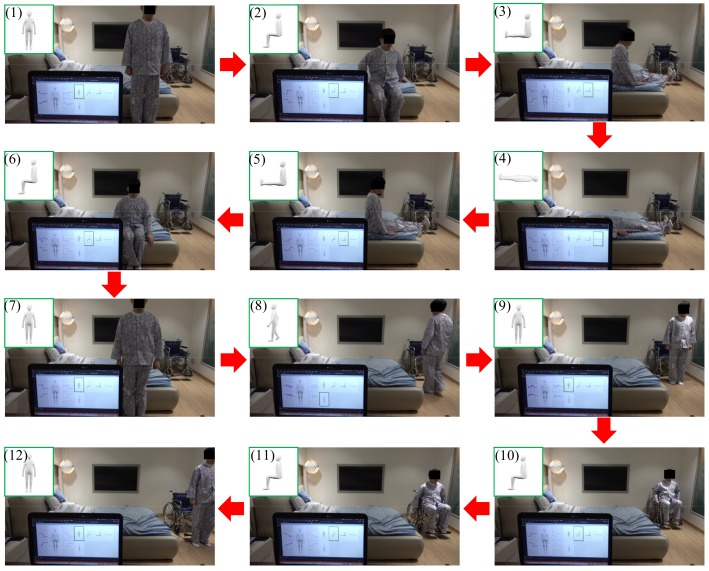
Test of the patient motion and posture monitoring system. (**1**–**7**) The patient lies down on the bed, and then stands up. (**7**–**9**) He moves to a wheelchair. (**9**–**12**) He sits on the wheelchair, moves via the wheelchair, and stands up from the wheelchair.

Figure 8 displays the captured computer screen image of the patient motion and posture monitoring system. At the left of the screen, it displays the processed values from the sensors of the left knee and hip and the right knee and hip. At the right of the screen, the detected patient posture is shown with a green outline. A demonstration of the patient motion and posture monitoring system is shown in Figure 9. Therein, the patient performs the transitions: (1) standing → (2) sitting on the bed → (3) sitting knee extension on the bed → (4) supine on the bed → (5) sitting knee extension on the bed → (6) sitting on the bed→ (7) standing up → (8) walking to the wheelchair → (9) stopping in front of the wheelchair → (10) sitting on the wheelchair→ (11) moving on the wheelchair → (12) standing up. All patient motion and posture monitoring of Figure 9 are displayed in Video S1 as Supplementary Material.

Additionally, we conduct gait detection tests from the other 10 users wearing the patient clothing. Specifically, the users walk and stop 50 times for a total of 1000 tests, 100 tests per user. We give the users simple orders about walking and stopping without specifying speed. Table 2 shows the success rates from the user test of the patient motion and posture monitoring system. The success rates are over 93%, and the total average is 97.5%. Several failed cases missed the changes or falsely detected sitting. In addition, most errors happen at taller users than the subject with the treadmill test. These errors can be attributed to the slow walking speeds of users and the low sensor outputs of the loose patient clothing. The success rate can be improved by selecting the gait detection frequency through personal statistics. For example, when we use the monitoring system for weak patients, they can have low walking speed. In this case, we need to adjust the gait detection frequency as lower value.

## 6. Conclusions

In this paper, we introduced a gait recognition system with flexible piezoelectric sensors in loose clothing. The flexible sensors were attached to the knee and hip parts on the pants. We analyzed the sensor outputs of a user during walking. Specifically, we investigated raw signals from the sensors, signal processed signals from integration, and FFT magnitude spectra. From the analysis, we found that the signals of the hip sensors are more suitable for gait detection. We demonstrated the gait detection method in a real-time patient motion and posture monitoring system. The monitoring system was utilized to detect five postures including walking and eight transitions between the postures. We showed the successful operation of the monitoring system in a demo video, which consisted of motions and postures around a bed and wheelchair. Additionally, our gait detection system was tested on 10 subjects, and the success rates were over 93%. The successful performance of our system shows the feasibility of gait detection from loose clothes using flexible sensors. We anticipate that the same methodology can be utilized to explore gait recognition using alternative flexible sensors. Moreover, it can provide an insight into motion recognition using smart suits or textiles.

## Figures and Tables

**Table 1:** Walking speeds, frequencies, and detection rate on the treadmill.

Speed [km/h]	Frequency [Hz]	Detection Rate [%]
0.5	0.25	19
1	0.41	36
2	0.61	69
3	0.79	98
4	0.92	100
5	1.04	100
6	1.14	100

**Table 2:** Success rate of the gait recognition in the patient motion and posture monitoring system.

Subject Number	Gender	Age [Years]	Weight [kg]	Height [cm]	Success Rate [%]
#1	M	25	60	174	96
#2	F	29	51	161	96
#3	M	26	70	175	100
#4	M	25	63	173	95
#5	M	33	77	166	100
#6	M	26	64	170	100
#7	M	25	70	177	93
#8	M	33	72	173	100
#9	M	28	68	178	95
#10	F	28	48	159	100

## Supplementary Materials

The following are available online at http://www.mdpi.com/1424-8220/18/2/468/s1.
